# Delegation of patient related tasks to allied health assistants: a time motion study

**DOI:** 10.1186/s12913-022-08642-7

**Published:** 2022-10-24

**Authors:** David A Snowdon, Olivia A King, Amy Dennett, Jo-Anne Pinson, Michelle M Shannon, Taya A Collyer, Annette Davis, Cylie M Williams

**Affiliations:** 1 Peninsula Clinical School, Central Clinical School, Monash University, Frankston, VIC Australia; 2 Academic Unit, Peninsula Health, Frankston, VIC Australia; 3 National Centre for Healthy Ageing, Melbourne, VIC Australia; 4 Monash Centre for Scholarship in Health Education, Monash University, Clayton, VIC Australia; 5 Allied Health Department, Barwon Health, Geelong, VIC Australia; 6 Allied Health Clinical Research Office, Eastern Health, Box Hill, VIC Australia; 7 School of Allied Health Human Services and Sport, La Trobe University, Bundoora, VIC Australia; 8 Medical Imaging Department, Monash Health, Clayton, VIC Australia; 9 Department of Medical Imaging and Radiation Sciences, Monash University, Clayton, VIC Australia; 10 Medical Imaging Department, Peninsula Health, Frankston, VIC Australia; 11 Allied Health Workforce Innovation Strategy Education Research (WISER) unit, Monash Health, Clayton, VIC Australia; 12 School of Primary and Allied Health Care, Monash University, Frankston, VIC Australia

**Keywords:** Healthcare Assistant, Healthcare Support Workers, Allied Health Occupations, Allied Health Professional, Delegation, Supervision, Time Motion Studies, Health Workforce

## Abstract

**Background:**

Allied health assistants (AHAs) are support staff who complete patient and non-patient related tasks under the delegation of an allied health professional. Delegating patient related tasks to AHAs can benefit patients and allied health professionals. However, it is unclear whether the AHA workforce is utilised optimally in the provision of patient care. The purpose of this study was to determine the proportion of time AHAs spend on patient related tasks during their working day and any differences across level of AHA experience, clinical setting, and profession delegating the task.

**Methods:**

A time motion study was conducted using a self-report, task predominance work sampling method. AHAs were recruited from four publicly-funded health organisations in Victoria, Australia. AHAs worked with dietitians, occupational therapists, physiotherapists, podiatrists, social workers, speech pathologists, psychologists, and exercise physiologists. The primary outcome was quantity of time spent by AHAs on individual task-categories. Tasks were grouped into two main categories: patient or non-patient related activities. Data were collected from July 2020 to May 2021 using an activity capture proforma specifically designed for this study. Logistic mixed-models were used to investigate the extent to which level of experience, setting, and delegating profession were associated with time spent on patient related tasks.

**Results:**

Data from 51 AHAs showed that AHAs spent more time on patient related tasks (293 min/day, 64%) than non-patient related tasks (167 min/day, 36%). Time spent in community settings had lower odds of being delegated to patient related tasks than time in the acute hospital setting (OR 0.44, 95%CI 0.28 to 0.69, P < 0.001). Time delegated by exercise physiologists and dietitians was more likely to involve patient related tasks than time delegated by physiotherapists (exercise physiology: OR 3.77, 95% 1.90 to 7.70, P < 0.001; dietetics: OR 2.60, 95%CI 1.40 to 1.90, P = 0.003). Time delegated by other professions (e.g. podiatry, psychology) had lower odds of involving patient related tasks than physiotherapy (OR 0.37, 95%CI 0.16 to 0.85, P = 0.02).

**Conclusion:**

AHAs may be underutilised in community settings, and by podiatrists and psychologists. These areas may be targeted to understand appropriateness of task delegation to optimise AHAs’ role in providing patient care.

**Supplementary Information:**

The online version contains supplementary material available at 10.1186/s12913-022-08642-7.

## Background

Allied health assistants (AHAs) support allied health professionals (AHPs) in clinical and administrative tasks under supervision or delegation.[1] Allied health is a broad term commonly describing non-nursing or medical professionals such as physiotherapy, podiatry, psychology, occupational therapy, speech pathology and social work.[2, 3] AHAs’ role complement allied health work and assist in the delivery of services across a range of clinical settings.[2] AHA qualifications vary from workplace training to certificate-level qualification.[4, 5].

Tasks performed by AHAs can be categorised as either patient or non-patient related. Non-patient related tasks include administration duties.[6, 7] Patient related tasks include any therapy that is provided to patients and may involve an AHA. They may assist AHPs in the provision of therapy or administer therapy independently.[6, 7] This form of patient related task is commonly categorised as ‘direct’ patient activity, as the patient is directly involved in the task. Patient related tasks can also be ‘indirect’, where the task focus is directed away from individual patient involvement but the task affects overall patient care. Indirect tasks include documenting patient care and arranging hire of equipment.

Delegation of tasks to AHAs is believed to benefit healthcare organisations, healthcare professionals and patients. A recent systematic review examining organisational and patient benefits of AHAs found AHA provision of dietetic/nutritional therapy reduces mortality following hip fracture.[8] Furthermore, AHA provision of exercise may increase the likelihood of patients discharging home from hospital compared to usual care.[8] It is also believed AHPs who delegate tasks to AHAs are available to carry out complex tasks,[9, 10] resulting in improved healthcare workforce capacity.[7].

The potential for AHAs to increase the capacity of allied health services is particularly appealing to the international allied health workforce, as it faces an increased demand from an ageing population with complex healthcare needs.[11, 12] In an attempt to unlock this potential, there has been significant investment in the assistant workforce to support allied health professionals working in Australia, Canada, Finland, New Zealand, Norway, the United Kingdom, and the United States of America.[8, 13–15] Across all countries there has been an emphasis on enhancing assistants’ roles in the provision of patient care. This highlights that delegating patient related tasks to AHAs is a priority for the allied health workforce in dealing with the ever-increasing demand for services.

Despite reported benefits of the AHA workforce, it is estimated up to one quarter of AHP time is spent on tasks that could be delegated to the AHA workforce, with most of these tasks involving direct patient care.[7, 16] Withholding delegation of patient related tasks to AHAs has been attributed to a number of factors, including an unwillingness to delegate tasks and lack of clarity about AHA scope of practice, roles and responsibilities.[4, 17] AHAs spend between 9% and 73% of their working day on direct patient care.[10, 18] However, previous studies have been limited to AHAs working with only one profession,[18] or in a single healthcare setting.[10, 18] Furthermore, it is not known what differences, if any, exist between AHAs with different levels of experience (e.g. junior/mid-level and advanced scope/senior AHA), who work in different settings (e.g. hospital, rehabilitation or community), or under delegation and supervision of different allied health professions. Understanding tasks AHAs routinely perform and any differences, may inform where AHAs are being underutilised in the provision of patient care. This information could help guide initiatives to enhance delegation of patient related tasks to AHAs, ultimately benefiting patient care and assisting in meeting the increased demand for healthcare.

Therefore, we conducted a time motion study to determine the proportion of time AHAs spend on patient related tasks during their working day and how this differs across level of AHA experience, setting, and profession delegating the task.

## Methods

### Study design

We conducted a time motion study to quantify the time AHAs spent on tasks.[19] The Suggested Time And Motion Procedures (STAMP) guidelines were followed during the study design and reporting.[19] Multi-site ethics approval was provided by the Monash Health Human Research Ethics Committee (Approval no. RES-20-0000118 L).

### Participants

Participants were AHAs working at one of four publicly-funded health organisations in Victoria, Australia. Three of these organisations were located in Melbourne, and one in regional Victoria. Participants included AHAs who worked within the Victorian government therapy grouping of allied health professions, including dietetics, occupational therapy, physiotherapy, podiatry, social work, speech pathology, psychology, and exercise physiology.[2, 20] All participants provided written informed consent.

### Outcomes

The primary outcome measure was quantity of time spent by AHAs on individual task-categories. Tasks were grouped into two main categories: patient or non-patient related activities. Patient related activities included any activity involved in the process of providing support (i.e. assessment, treatment, clinical reporting, or equipment provision to patients). Non-patient related activities included those not involved in the process of providing patient support, but rather supporting the allied health department and/or their staff (i.e. department equipment provision, supervision of AHAs, research or quality activities, administration, transition between tasks).

Patient related activities were further classified as either direct or indirect patient activities. Direct patient activities involved provision of care and communication with the patient (e.g. assessment, treatment, provision of equipment). Indirect patient activities included activities not involving patient communication, but were another component of service delivery (e.g. clinical documentation, clinical handover, arranging hire of equipment).

### Data collection

Participants were recruited via email to AHA distribution lists and face-to-face at department staff meetings. We used a self-report, task predominance work sampling method, where participating AHAs recorded the predominant task they performed over a 10-minute interval on an activity capture proforma specifically designed for this study.[19, 21, 22] The task codes used on the proforma were based on patient and non-patient related tasks previously established from focus groups with AHAs and AHPs (Additional File 1).[7, 23] These codes were also classified into 10 broad categories: assessment, treatment, complex cases, clinical reporting, discharge planning, equipment and environment, supervision, research and quality, administration, and transition between tasks (i.e. walking, driving, waiting).[7] A description of tasks included in each code category is provided in Table 1. Additional data about the task were collected, including the profession delegating the task, mode of communication during task, and the clinical stream and location the task was performed.

**Table 1:** Task categories

Task category	Description
Assessment	Tasks that involve assisting with assessment of patient performance or health. This may include objective assessment (e.g. walking speed, performance of activities of daily living, feeding) or subjective assessment (e.g. asking patients to report on their health/function).
Treatment	Any form of therapy provided to the patient including education.
Complex cases	Tasks that require specialist training or oversight to provide (e.g. assisting physiotherapist with patients who require two people to transfer, inspection/management of scars, monitor a patient’s splint).
Clinical reporting	Any form of clinical documentation or clinical handover to allied health, nursing or medical professionals.
Discharge planning	Assisting with discharge planning processes, including sourcing accommodation vacancies, organising support services, organising follow-up community therapy, assisting with home visit, and assisting with referrals.
Equipment and environment	Provision or maintenance of equipment including wheelchairs, frames, pressure care and other assist devices. This also includes ordering clinical equipment for the department and maintenance of the clinical environment (e.g. cleaning equipment, testing hydrotherapy pool chemistry).
Supervision	Tasks that involve supervision/training of AHAs, staff or students, or attending own supervision/training.
Research and quality	Any task related to research or quality improvement projects.
Administration	Any non-clinical administration task (e.g. organising outpatient appointments, engineering requests, typing meeting minutes, attendance at department meetings).
Transition between tasks	Travel between tasks (i.e. walking, driving) or time spent waiting in-between delegated tasks

AHA demographics were also collected. This included level of experience, measured using the health care industry applied pay grade, categorised as grade 2 (junior/mid-level) or grade 3 (advanced scope of practice/senior), and clinical setting (i.e. acute hospital, sub-acute hospital or community healthcare). Qualifications and time spent working as an AHA were also collected.

The proforma was piloted by three AHAs, one each working in acute, sub-acute and community, prior to study commencement. Their feedback led to further development of the final version. The final proforma is provided in Additional File 2.

AHAs completed the tool for two working days on separate days of the week (e.g. Monday and Wednesday) to ensure that data collected were representative of the AHAs’ usual tasks across the week. AHAs were requested to provide data for their whole working day with the exception of ‘break times’ (e.g. morning tea, lunch). If data were missing or if any further clarification was required on the tasks that were documented, AHAs were contacted by researchers to obtain/clarify this data. Following data entry, AHAs’ workday activity were non-identifiable and were not shared with their manager or organisation. Data were collected from July 2020 to May 2021.

### Statistical analysis

Average time spent on tasks per day (minutes/day) and proportion of time spent on task-categories (%) were used to describe the data. To investigate the extent to which AHA grade, setting, and delegating profession were associated with task-category, two logistic mixed-models were fit at the timepoint level to compare (i) the odds of patient vs. non-patient tasks, and (ii) direct vs. indirect patient tasks. Standard logistic regression modelling was inappropriate for these data due to the strong correlation across timepoints for individual AHAs.[24] Our mixed models accounted for random effects due to individual AHAs, and also due to days within individuals. In this way, the tendency for individual AHAs to report similar data each day is appropriately managed. Integration (estimation of log-likelihood) was achieved via mean-variance adaptive Gauss–Hermite quadrature. All analyses were completed using STATA Version 16.0 (StataCorp, College Station, Tx, USA).

## Results

### Participants

Fifty-one AHAs participated in the study and provided time motion data for two full working days totaling 46,890 min of total activity (4,689 data points). Table 2 reports the AHA characteristics.

**Table 2:** Allied Health Assistant (AHA) characteristics (n = 51)

Characteristic	n (%)
**Grade**	
2 (junior/mid-level)	19 (37)
3 (advanced scope of practice)	32 (63)
**Clinical setting**	
Acute hospital	12 (24)
Community health service	17 (33)
Sub-acute hospital	21 (41)
Sub-acute and community joint role	1 (2)
**Profession delegating**	
Dietetics	1 (2)
Multi-disciplinary	17 (33)
Occupational therapy	6 (12)
Physiotherapy	23 (45)
Social work	1 (2)
Speech pathology	3 (6)
**Qualification**	
Bachelor of Human Movement	1 (2)
Bachelor of Nursing	1 (2)
Bachelor of Physiotherapy (International)^A^	2 (4)
Certificate 3 Allied Health Assistance	8 (16)
Certificate 4 Allied Health Assistance	32 (63)
Master of Applied Science	1 (2)
Non-health qualification	3 (6)
No qualification	3 (6)
**Time spent working as an AHA** ^**B**^ **(years)**	10 (7.5)

A - Not deemed substantially equivalent qualification for physiotherapy registration in Australia; B – mean (sd)

### Time spent on tasks

On average, AHAs spent more time on patient related tasks (293 min/day, 64%) than non-patient related tasks (167 min/day, 36%). Of the time spent on patient related tasks, AHAs spent more time on direct patient tasks (197 min/day, 67%) than indirect patient tasks (96 min/day, 33%). The time spent on task categories is summarised in Table 3 and stratified by AHA grade, clinical setting and profession delegating in Additional File 3. A summary of communication tasks, and the location and clinical stream in which patient related tasks were performed is provided in Additional File 4.

**Table 3:** Time spent on task categories

Task	Minutes/day (%)
**Overall**	
Patient Tasks	293 (64)
Non-patient	167 (36)
**Patient Tasks**	
Direct	197 (43)
Indirect	96 (21)
**Task Categories**	
Treatment	157 (34)
Clinical reporting	78 (17)
Administration	76 (17)
Equipment/Environment	65 (14)
Complex cases	26 (6)
Supervision	21 (5)
Transition	15 (3)
Discharge planning	8 (2)
Assessment	8 (2)
Research/Quality	7 (2)

### Factors associated with time spent on patient related tasks

Results of regression models are presented in Table 4. Following adjustment for AHA grade, delegating profession and random effects, time spent in the community setting had lower odds of being delegated to patient related tasks than time in the acute hospital setting (OR 0.44, 95%CI 0.28 to 0.69, P < 0.001). In the same model, exercise physiologists and dietitians were more likely to delegate time for patient-related tasks than physiotherapists (exercise physiology: OR 3.77, 95% 1.90 to 7.70, P < 0.001; dietetics: OR 2.60, 95%CI 1.40 to 1.90, P = 0.003). Time delegated by other professions (i.e. podiatry and psychology) had lower odds of involving patient related tasks than physiotherapy (OR 0.37, 95%CI 0.16 to 0.85, P = 0.02).

**Table 4:** Factors influencing time spent on patient related (vs. non-patient related) tasks and direct patient (vs. indirect) tasks

Variable		Patient Related Tasks 4,689 total timepoints			Direct Patient Tasks 2,989 total timepoints	
		Odds Ratio (95%CI)	p		Odds Ratio (95%CI)	P
**AHA grade**						
Grade 2		Reference group			Reference group	
Grade 3		0.87 (0.61 to 1.24)	0.445		0.67 (0.43 to 1.05)	0.082
**Clinical setting**						
Acute		Reference Group			Reference Group	
Community		0.44 (0.28 to 0.69)*	**< 0.001**		1.72 (0.98 to 3.01)	0.061
Subacute		0.92 (0.59 to 1.43)	0.718		1.75 (1.01 to 3.00)*	**0.045**
**Profession delegating**						
PT		Reference group			Reference group	
DT		2.60 (1.40 to 1.90) *	**0.003**		1.38 (0.63 to 3.00)	0.427
EP		3.77 (1.90 to 7.70) *	**< 0.001**		1.09 (0.41 to 2.90)	0.864
OT		0.85 (0.62 to 1.15)	0.299		0.58 (0.39 to 0.88)*	**0.010**
SW		0.60 (0.34 to 1.00)	0.069		0.07 (0.02 to 0.18)*	**< 0.001**
SP		0.96 (0.60 to 1.60)	0.878		1.39 (0.70 to 2.75)	0.350
Other		0.37 (0.16 to 0.85) *	**0.019**		1.40 (0.44 to 2.90)	0.568

AHA – Allied Health Assistant; DT – Dietetics; EP – Exercise Physiology; OT – Occupational Therapy; PT – Physiotherapy; SP – Speech Pathology; SW – Social Work; * - statistically significant. NB: ‘Other’ includes podiatry and psychology.

### Factors associated with time spent on direct patient tasks

Following adjustment for AHA grade, delegating profession and random effects, AHA time spent in sub-acute hospital settings had higher odds of involving direct patient tasks than the acute hospital setting (OR 1.75, 95%CI 1.01 to 3.00, P = 0.045) (Table 4). Social workers and occupational therapists were less likely to delegate time for direct patient tasks than physiotherapists (social work: OR 0.07, 95%CI 0.02 to 0.18, P < 0.001; occupational therapy: OR 0.58, 95%CI 0.39 to 0.88, P = 0.01).

## Discussion

To the best of our knowledge, this is the first study using a time motion design to quantify the role of AHAs who work with a variety of allied health professions and across multiple healthcare settings. AHAs spent approximately two thirds of their working day on patient related tasks, with the majority of this time spent on direct patient tasks. The proportion of time AHAs spent on patient related and direct patient tasks differed across clinical setting and profession delegating the task. This highlights that AHAs may be underutilised within the Australian health system and there could be opportunities to expand their role in patient care. International comparative data is required to understand how broad this issue may be.

In recent years, health policy in Australia has prioritised delegation of patient tasks to AHAs to improve the reach and efficiency of allied health care.[4, 7, 16] This has been challenging due to AHPs’ reluctance to delegate clinical tasks, in part due to uncertainty about the role of AHAs.[1, 4, 6] Victorian government led initiatives are aiming to alleviate this uncertainty, such as the development and implementation of frameworks to inform AHA scope of practice.[23, 25] These initiatives have encouraged expansion of AHAs’ scope of practice and greater utilisation of AHAs by professions other than physiotherapy and occupational therapy.[26, 27] Our findings potentially reflect that AHAs working in the Victorian public healthcare setting are well supported to undertake a predominantly patient-facing role.

Previous evaluations of the AHA role have shown that the time they spend providing patient care is variable. Physiotherapy assistants working in a community health setting in the United Kingdom spent less time on direct patient activities than the AHAs in our cohort, with only 9–16% of their working day dedicated to direct patient care.[18] These assistants had a greater non-clinical administrative role than the assistants in our study, which accounted for 65–78% of their working day and mostly consisted of ‘data inputting’.[18] Rehabilitation assistants, who assist both allied health and nursing professionals in a rehabilitation unit in the United Kingdom spent 73% of their working day on direct patient care.[10] This is 30% greater than the amount of time that AHAs in our study spent on direct patient care and likely due to the additional role supporting nursing professionals (e.g. washing and dressing patients) and that these assistants were not delegated, or did not report, clinical reporting tasks (e.g. clinical documentation).[10] Therefore, there may be opportunities to further expand Australian AHAs role in the provision of patient care, and our results reveal the specific clinical settings and allied health professions that could be targeted to understand appropriateness of task delegation to optimise utilisation of the AHA workforce for providing patient care.

Administrative tasks are important for the operation of allied health departments, however delegation of these tasks to administration staff may provide more opportunities for AHAs to further engage in patient related tasks. In our study administration tasks accounted for 14–21% of AHAs’ working day, with AHAs working in community settings spending the highest proportion of time on these tasks. Delegating patient related tasks to AHAs can be challenging in community settings because funding is activity-based and allocated to professions based on provided interventions.[28] Given that AHAs are not qualified to provide all of these interventions, delegation of patient tasks to AHAs can fluctuate. Furthermore, allied health services are appointment-based and AHAs are likely to be delegated administration tasks when appointments are unfilled or during patient non-attendance. However, these challenges are not insurmountable and allied health departments should strive to use AHAs to their full scope of practice.

Delegation of patient related tasks to AHAs may be challenging for the allied health professions with a smaller workforce (e.g. podiatry or psychology). Smaller professions may have little experience delegating tasks to AHAs and likely still establishing capability of the AHA workforce to perform patient related tasks specific to their profession.[6, 29] Accommodating the time investment required to train AHAs to perform patient related tasks is difficult with a small workforce and competing clinical demands.[29] Ongoing experience working with AHAs and access to educational resources to assist with upskilling AHAs (e.g. external vocational courses), may facilitate delegation of patient tasks by the professions with a small workforce.

Our study indicates that AHAs working in the acute setting spend the lowest proportion of their day engaging independently in direct patient tasks. This adds further weight to arguments for the expansion of AHAs’ roles in acute hospital settings.[13, 30] AHAs’ comparative lack of independence in the acute setting is likely a reflection of their role in assisting AHPs with complex cases and the risk associated with treating people who are acutely unwell.[13] While hesitation to delegate care of acutely unwell patients may be warranted, evidence suggests that AHAs can provide safe and effective care in the acute hospital setting.[8, 13, 31] However, the training and supervision/governance processes required for safe delegation in the acute setting are not clear, and further research and resources are needed to establish these processes.[8, 13, 32].

Variation in delegation of patient related tasks and direct patient tasks may also be warranted. For example, it may be reasonable that occupational therapists delegate less direct patient tasks than physiotherapists because the occupational therapy profession has a greater role in the prescription of equipment.[33] This is an important role in allied health care and should not be devalued; ultimately it contributes to meeting the increased demand for healthcare. The results from our study highlight where AHAs may potentially be underutilised, and where further investigation of their role in patient care is warranted to determine whether the delegation of patient related tasks can be enhanced.

Our results may also guide what forms of patient care could most readily be delegated to AHAs. We found that AHAs spent most time providing therapy to geriatric, orthopaedic and neurological patient populations in ward or dedicated therapy areas. Therefore, initiatives aimed at increasing delegation of patient care to AHAs may be best targeted to these populations and environments, as they are likely to require less training and upskilling of AHAs. In comparison, delegating the care of more specialist patient populations (e.g. paediatrics, oncology, women’s health) or care that is provided in environments that are not conducive to direct supervision of AHAs (e.g. patient’s home) may require greater investment in training and upskilling of AHAs and the development of competencies specific to these areas of practice.[34, 35].

A strength of our study was the use of a proforma informed by previous research about AHAs’ roles.[7, 23] This ensured consistency in task definitions and data collection. This proforma should be considered in similar studies or where organisations are seeking to make changes in the structure and delegations of AHAs. Limitations must also be considered when interpreting our results. First, our study included AHAs working for Victorian publicly-funded health organisations and results may not be generalisable to other Australian or international healthcare settings. Due to COVID-19 social distancing measures direct observation of AHA activity was not feasible, as such we were unable to check the fidelity of self-reported activity by directly observing participants. Self-report work sampling may have led to overestimates of productive work activities by AHAs.[36] However, participants were assured that their workday activity were non-identifiable and would not be shared with their manager or organisation. By conducting our analysis at the timepoint level, we have estimated the influence of characteristics (i.e. clinical setting, profession delegating, AHA experience) on whether delegated time is assigned to patient or non-patient tasks. A limitation of this design is that we cannot make formal statements regarding differences between types of AHA. This would require a larger sample of AHAs. Last, COVID-19 social distancing measures may have impacted AHAs’ usual role in some direct patient tasks, such as group therapy.

## Conclusion

AHAs perform a key role in the provision of patient care, spending most of their working day on patient related activities. However, the proportion of time they spend on patient related tasks varies across clinical settings and allied health professions. In particular, our findings indicated that AHAs working in Australia may be underutilised in community settings, and by podiatrists and psychologists. Future initiatives could target these areas to understand appropriateness of task delegation to optimise utilisation of the AHA workforce for providing patient care. Future initiatives could also focus on understanding the roles and scope of the international AHA workforce.

## Electronic supplementary material

Below is the link to the electronic supplementary material.

Supplementary Material 1

Supplementary Material 2

Supplementary Material 3

Supplementary Material 4

## Data Availability

The data generated and analysed during the current study are not publicly available as participants did not provide consent for data sharing, however de-identified data is available from the corresponding author on reasonable request.

## List of abbreviations

AHA: allied health assistant
AHP: allied health professional
