# Supplementary material for: Delegation of patient related tasks to allied health assistants: a time motion study
Source: BMC Health Serv Res. 2022 Oct 24;22:1280. doi: 10.1186/s12913-022-08642-7 (PMC9590386; doi:10.1186/s12913-022-08642-7)
Supplement: Supplementary file 1 — Supplementary Material 1 [file 12913_2022_8642_MOESM1_ESM.docx]

**Additional File 1**: Task Codes

| **Code for Task** | **Code** |
| --- | --- |
| **Assessment** | |
| Outcome measurement | FA1 |
| Subjective assessment | FA2 |
| Other (comment on activity capture proforma) | FA3 |
| **Treatment** | |
| Education/Information provision | FT1 |
| Balance exercise | FT2 |
| Mobility training | FT3 |
| Hydrotherapy exercise | FT4 |
| General exercise (e.g. strengthening) | FT5 |
| Chest/Airway clearance | FT6 |
| Portering to/from gym | FT7 |
| Activities of daily living – personal care | FT8 |
| Activities of daily living – domestic activities | FT9 |
| Activities of daily living – community activities | FT10 |
| Relaxation /energy conservation | FT11 |
| Pressure area care | FT12 |
| Speech therapy | FT13 |
| Swallowing therapy | FT14 |
| Provision of thickened fluid | FT15 |
| Foot care – wound care | FT16 |
| Foot care – toe nail care | FT17 |
| Provision of supplements | FT18 |
| Menu selection | FT19 |
| Upper limb exercise | FT20 |
| Other (comment on activity capture proforma) | FT21 |
| **Complex cases** | |
| Doubles (assist with two person assist treatments) | FC1 |
| Other (comment on activity capture proforma) | FC2 |
| **Clinical reporting** | |
| Documentation in progress notes/exercise sheets | FR1 |
| Clinical handover | FR2 |
| Reading notes (preparation for treatment) | FR3 |
| Team meeting attendance (patient care discussed) | FR4 |
| Other (comment on activity capture proforma) | FR5 |
| **Discharge Planning** | |
| Assist transition to residential care (source accommodation vacancies) | FD1 |
| Assist with organising support services on discharge | FD2 |
| Assist with organising follow up therapy on discharge | FD3 |
| Assist with home visit (attend with therapist) | FD4 |
| Assist with written or electronic referrals | FD5 |
| Other (comment on activity capture proforma) | FD6 |

| **Code for Task** | **Code** |
| --- | --- |
| **Equipment and environment** | |
| Equipment provision and education | FE1 |
| Arranging purchase/hire of equipment | FE2 |
| Wheelchair provision/education/fitting | FE3 |
| Clean equipment / infection control / maintain equipment | FE4 |
| Equipment ordering for department | FE5 |
| Preparing area for treatment (including home visit) | FE6 |
| Hydrotherapy chemical/pool testing | FE7 |
| Other (comment on activity capture proforma) | FE8 |
| **Supervision** | |
| Supervising AHAs (supervisor) | FS1 |
| Supervising students (supervisor) | FS2 |
| Attending own supervision (supervisee) | FS3 |
| Other (comment on activity capture proforma) | FS4 |
| **Research and quality** | |
| Research and quality | FQ1 |
| **Administration** | |
| Photocopying /laminating/filing/scanning | GA1 |
| Timetabling / organising outpatient appointments | GA2 |
| Wait list management | GA3 |
| Attendance at staff/workload meeting | GA4 |
| Billing | GA5 |
| Engineering requests/room booking | GA6 |
| Typing up meeting minutes/ correspondence | GA7 |
| Statistics | GA8 |
| Other (comment on activity capture proforma) | GA9 |
| **Other** | |
| Comment on spreadsheet (comment on activity capture proforma) | FO1 |

| **Code for Transition** | **Code** |
| --- | --- |
| Travel - Walking | H1 |
| Travel - Car | H2 |
| Waiting | H3 |
