# Supplementary material for: Delegation of patient related tasks to allied health assistants: a time motion study
Source: BMC Health Serv Res. 2022 Oct 24;22:1280. doi: 10.1186/s12913-022-08642-7 (PMC9590386; doi:10.1186/s12913-022-08642-7)
Supplement: Supplementary file 2 — Supplementary Material 2 [file 12913_2022_8642_MOESM2_ESM.docx]

**Additional File 2**. Activity Capture Proforma

| **Rostered Hours for today** | | **Patient/Client Count** | |
| --- | --- | --- | --- |
| **AHA Grade level** | **AHA years of experience** | **AHA qualification** | **A: Clinical Setting**  **(acute, sub-acute, community)** |

| Time | B  Profession delegating | C  Clinical stream | D  Communication | E  Location | F  Task | G  Transition |
| --- | --- | --- | --- | --- | --- | --- |
| 8:00-8:10 |  |  |  |  |  |  |
| -8:20 |  |  |  |  |  |  |
| -8:30 |  |  |  |  |  |  |
| -8:40 |  |  |  |  |  |  |
| -8:50 |  |  |  |  |  |  |
| -9:00 |  |  |  |  |  |  |

| Time | B  Profession delegating | C  Clinical stream | D  Communication | E  Location | F  Task | H  Transition |
| --- | --- | --- | --- | --- | --- | --- |
| -9:10 |  |  |  |  |  |  |
| -920 |  |  |  |  |  |  |
| -9:30 |  |  |  |  |  |  |
| -9:40 |  |  |  |  |  |  |
| -9:50 |  |  |  |  |  |  |
| -10:00 |  |  |  |  |  |  |
| -10:10 |  |  |  |  |  |  |
| -10:20 |  |  |  |  |  |  |
| -10:30 |  |  |  |  |  |  |
| -10:40 |  |  |  |  |  |  |
| -10:50 |  |  |  |  |  |  |
| -11:00 |  |  |  |  |  |  |
| -11:10 |  |  |  |  |  |  |
| -11:20 |  |  |  |  |  |  |
| -11:30 |  |  |  |  |  |  |
| -11:40 |  |  |  |  |  |  |
| -11:50 |  |  |  |  |  |  |
| -12:00 |  |  |  |  |  |  |
| -12:10 |  |  |  |  |  |  |
| -12:20 |  |  |  |  |  |  |
| -12:30 |  |  |  |  |  |  |
| -12:40 |  |  |  |  |  |  |
| -12:50 |  |  |  |  |  |  |
| -13:00 |  |  |  |  |  |  |
| -13:10 |  |  |  |  |  |  |
| -13:20 |  |  |  |  |  |  |
| -13:30 |  |  |  |  |  |  |
| -13:40 |  |  |  |  |  |  |
| -13:50 |  |  |  |  |  |  |
| -14:00 |  |  |  |  |  |  |
| -14:10 |  |  |  |  |  |  |
| -14:20 |  |  |  |  |  |  |
| -14:30 |  |  |  |  |  |  |
| -14:40 |  |  |  |  |  |  |
| -14:50 |  |  |  |  |  |  |
| -15:00 |  |  |  |  |  |  |
| -15:10 |  |  |  |  |  |  |
| -15:20 |  |  |  |  |  |  |
| -15:30 |  |  |  |  |  |  |
| -15:40 |  |  |  |  |  |  |
| -15:50 |  |  |  |  |  |  |
| -16:00 |  |  |  |  |  |  |
| -16:10 |  |  |  |  |  |  |
| -16:20 |  |  |  |  |  |  |
| -16:30 |  |  |  |  |  |  |
| -16:40 |  |  |  |  |  |  |
| -16:50 |  |  |  |  |  |  |
| -1700 |  |  |  |  |  |  |
