# Supplementary material for: Delegation of patient related tasks to allied health assistants: a time motion study
Source: BMC Health Serv Res. 2022 Oct 24;22:1280. doi: 10.1186/s12913-022-08642-7 (PMC9590386; doi:10.1186/s12913-022-08642-7)
Supplement: Supplementary file 3 — Supplementary Material 3 [file 12913_2022_8642_MOESM3_ESM.docx]

**Additional File 3**. Proportion of time spent on tasks by allied health assistant (AHA) grade, clinical setting and profession delegating

|  |  | **AHA grade** | |  | **AHA clinical setting** | | |  | **Profession delegating to AHA** | | | | | | |  | |
| --- | --- | --- | --- | --- | --- | --- | --- | --- | --- | --- | --- | --- | --- | --- | --- | --- | --- |
| **Tasks** |  | **Grade 2** | **Grade 3** |  | **Acute** | **Community** | **Sub-Acute** |  | **DT** | **EP** | **OT** | **PT** | **SW** | **SP** | **Other** | |  |
| **Overall** |  |  |  |  |  |  |  |  |  |  |  |  |  |  |  | |  |
| Time delegated, *mins* |  | 17420 | 29470 |  | 10770 | 17130 | 18990 |  | 1910 | 1420 | 8250 | 29610 | 1560 | 3470 | 670 | |  |
| Patient, *%* |  | 64 | 64 |  | 69 | 56 | 68 |  | 70 | 70 | 63 | 65 | 53 | 59 | 54 | |  |
| Non-patient, *%* |  | 36 | 36 |  | 31 | 45 | 32 |  | 30 | 30 | 37 | 35 | 47 | 41 | 46 | |  |
| **Patient Tasks** |  |  |  |  |  |  |  |  |  |  |  |  |  |  |  | |  |
| Time delegated, *mins* |  | 11140 | 18750 |  | 7430 | 9500 | 12960 |  | 1330 | 1000 | 5160 | 19170 | 820 | 2050 | 360 | |  |
| Direct patient, *%* |  | 73 | 63 |  | 58 | 71 | 71 |  | 58 | 71 | 60 | 73 | 10 | 58 | 70 | |  |
| Indirect patient, *%* |  | 27 | 37 |  | 42 | 29 | 29 |  | 42 | 29 | 40 | 27 | 90 | 42 | 30 | |  |
| **Task Categories** |  |  |  |  |  |  |  |  |  |  |  |  |  |  |  | |  |
| Time delegated, *mins* |  | 17420 | 29470 |  | 10770 | 17130 | 18990 |  | 1910 | 1420 | 8250 | 29610 | 1560 | 3470 | 670 | |  |
| Administration, *%* |  | 17 | 16 |  | 15 | 21 | 14 |  | 12 | 20 | 15 | 16 | 21 | 22 | 12 | |  |
| Assessment, *%* |  | <1 | 2 |  | 1 | 2 | 2 |  | 19 | 4 | 1 | 1 | 0 | <1 | 9 | |  |
| Clinical reporting, *%* |  | 14 | 19 |  | 20 | 14 | 18 |  | 30 | 20 | 19 | 15 | 10 | 24 | 13 | |  |
| Complex cases, *%* |  | 5 | 6 |  | 13 | 3 | 4 |  | 0 | 0 | <1 | 8 | 0 | 4 | 0 | |  |
| Discharge planning, *%* |  | <1 | 3 |  | 6 | 1 | <1 |  | 0 | 0 | 0 | 1 | 37 | 1 | 0 | |  |
| Equip/Environ, *%* |  |  |  |  |  |  |  |  |  |  |  |  |  |  |  | |  |
| Patient |  | 5 | 3 |  | 6 | 2 | 4 |  | 1 | 1 | 10 | 3 | 0 | 2 | 6 | |  |
| Non-patient |  | 13 | 9 |  | 9 | 11 | 10 |  | 5 | 7 | 11 | 12 | 5 | 7 | 3 | |  |
| Research/Quality, *%* |  | 1 | 2 |  | 1 | 2 | 1 |  | 0 | 0 | 2 | 1 | 3 | 0 | 31 | |  |
| Supervision, *%* |  | 2 | 6 |  | 5 | 5 | 4 |  | 7 | 3 | 6 | 3 | 17 | 9 | 0 | |  |
| Transition, *%* |  | 3 | 3 |  | 2 | 6 | 2 |  | 5 | 6 | 4 | 3 | 0 | 4 | 0 | |  |
| Treatment, *%* |  | 39 | 3 |  | 24 | 34 | 40 |  | 21 | 47 | 32 | 37 | 5 | 30 | 25 | |  |

Equip/environ – equipment/environment; DT – Dietetics; EP – Exercise Physiology; OT – Occupational Therapy; PT – Physiotherapy; SP – Speech Pathology; SW – Social Work.

NB: ‘Other’ includes podiatry and psychology.
