# Supplementary material for: Delegation of patient related tasks to allied health assistants: a time motion study
Source: BMC Health Serv Res. 2022 Oct 24;22:1280. doi: 10.1186/s12913-022-08642-7 (PMC9590386; doi:10.1186/s12913-022-08642-7)
Supplement: Supplementary file 4 — Supplementary Material 4 [file 12913_2022_8642_MOESM4_ESM.docx]

**Additional File 4**. Communication tasks, location and clinical stream

| **Variables** | **mins/day (%)**  n=51 |
| --- | --- |
| **Communication** |  |
| Total | 460 (100) |
| Face to face | 303 (66) |
| Videoconferencing | 53 (12) |
| Phone | 23 (5) |
| Email | 18 (4) |
| No communication | 63 (14) |
| **Location** |  |
| Total | 460 (100) |
| Therapy area (e.g. gym) | 146 (32) |
| Office | 145 (32) |
| Ward | 124 (27) |
| Other | 31 (7) |
| Patient’s home | 10 (2) |
| Missing | 3 (1) |
| **Clinical stream^A^** |  |
| Total^B^ | 293 (100) |
| Geriatric medicine | 121 (41) |
| Neurological | 72 (25) |
| Orthopaedic | 61 (21) |
| Oncology | 24 (8) |
| Cardiorespiratory | 20 (7) |
| Paediatrics | 14 (5) |
| Women’s health | 2 (1) |

A – can include multiple clinical streams per task (e.g. group therapy) and values may total >100% time spent on patient related activities; B – total includes only time spent on patient related tasks.
